# Clinical effectiveness of cell therapies in patients with chronic liver disease and acute-on-chronic liver failure: a systematic review protocol

**DOI:** 10.1186/s13643-016-0277-6

**Published:** 2016-06-14

**Authors:** Nwe Ni Than, Claire L. Tomlinson, Debashis Haldar, Andrew L. King, David Moore, Philip N. Newsome

**Affiliations:** National Institute for Health Research (NIHR) Birmingham Liver Biomedical Research Unit and Centre for Liver Research, University of Birmingham, Edgbaston, Birmingham, B15 2TT UK; Birmingham Clinical Trials Unit, Institute of Applied Health Research, University of Birmingham, Edgbaston, Birmingham, B15 2TT UK; Institute of Applied Health Research, University of Birmingham, Edgbaston, Birmingham, B15 2TT UK

**Keywords:** Chronic liver disease, Acute on chronic liver failure, Model for end-stage liver disease, Survival, Quality of life, Cell therapy, Granulocyte colony-stimulating factor

## Abstract

**Background:**

Chronic liver disease (CLD) is a major health burden worldwide. Liver cirrhosis, a form of CLD is the fifth most common cause of death in the UK. Acute-on-chronic liver failure (ACLF) is the result of an acute insult superimposed on patients with liver cirrhosis as a result of precipitating events such as infection or bleeding. ACLF has a high associated mortality as a result of multi-organ failure. The only effective treatment for CLD is liver transplantation, but the treatment is limited by shortage of donor organs. As a result, alternative treatments such as cell therapies have been studied in patients with liver diseases. This study will systematically review the evidence on clinical effectiveness of cell therapies in patients.

**Methods:**

All types of study design that investigate the effectiveness of cell therapies (haematopoietic, mesenchymal and unsorted cell types) of autologous or allogeneic origin and/or the use of granulocyte colony-stimulating factor in patients with CLD including ACLF will be included (except case reports). Both autologous and allogenic cell types will be included. The primary outcomes of interest are survival, model for end-stage liver disease score, quality of life and adverse events. Secondary outcomes include liver function tests, Child-Pugh score and events of liver decompensation. A literature search will be conducted in the following databases: MEDLINE, MEDLINE in Process, EMBASE and Cochrane Library (CENTRAL, CDSR, DARE, HTA databases). Trial registers will be searched for ongoing trials, as will conference proceedings. Reference lists of relevant articles and systematic reviews will be screened. Randomised controlled trial (RCT) evidence is likely to be scant; therefore, controlled trials and concurrently controlled observational studies will be primarily analysed and uncontrolled observational studies will be analysed where primary outcomes are not reported in the control studies or where uncontrolled studies have longer follow-up. Initial screening of studies will be carried by one reviewer with a proportion checked by another reviewer. Full-text selection will be performed by two reviewers independently against the pre-defined selection criteria. The data collection and the risk of bias assessment will be completed by one reviewer and counter checked by another reviewer for all selected studies. Where appropriate, data will be meta-analysed for each study design, therapy and outcome. Data specifically on ACLF will be treated as a subgroup.

**Discussion:**

This systematic review will identify the available evidence on the effectiveness of cell therapies in patients with CLD and in ACLF subgroup. The findings will aid decision-making by clinicians and health service leaders.

**Systematic review registration:**

PROSPERO CRD42016016104

**Electronic supplementary material:**

The online version of this article (doi:10.1186/s13643-016-0277-6) contains supplementary material, which is available to authorized users.

## Background

### Introduction on the underlying disease

Chronic liver disease (CLD) is a major health burden worldwide, with 29 million people in Europe affected by this condition [1]. Liver cirrhosis, also known as end-stage CLD, is a slow progressive disease in which normal liver tissue is replaced by fibrous tissue as a result of injury such as alcohol excess or viral hepatitis. Liver cirrhosis is the fifth most common cause of death in England and Wales after heart disease, cancer, stroke and respiratory disease [2]. Mortality from patients with liver cirrhosis is rising, and it is expected that it will double in the next 20 years [2]. Mortality is usually a result of complications of liver cirrhosis or from liver cancer/hepatocellular carcinoma (HCC). Complications of liver cirrhosis (known as decompensation) present as an intermittent or persistent altered mental state (hepatic encephalopathy), or manifestations of portal hypertension such as fluid accumulation in the abdomen (ascites), or bleeding from porto-systemic collateral varices. Liver cirrhosis is a recognised risk factor for the development of HCC. HCC is the fifth most common cause of cancer in Europe and constitutes 70–90 % of all cases of primary liver cancer [2]. Common causes of liver cirrhosis are listed in Table 1.

**Table 1:** Causes of chronic liver disease

Alcohol excess
Viral hepatitis (B and C)
Non-alcoholic fatty liver disease
Autoimmune mediated
• Autoimmune hepatitis
• Primary biliary cholangitis
• Primary sclerosing cholangitis
Genetic causes
• Alpha 1 antitrypsin deficiency
• Genetic haemochromatosis
• Wilson’s disease
Vascular aetiology
• Budd-Chiari syndrome

**Fig. 1:**
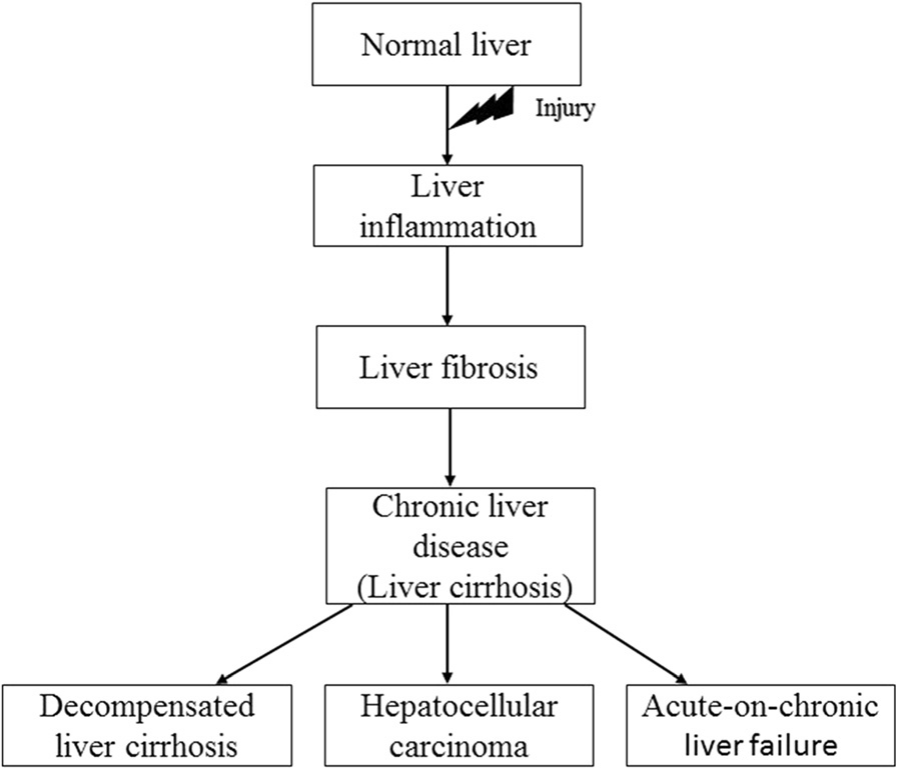
Clinical progression of liver disease

Acute-on-chronic liver failure (ACLF) is an acute deterioration of patients with liver cirrhosis that is precipitated by a physiological insult (e.g. infection) [3, 4]. It carries a worse prognosis than un-triggered decompensation of liver cirrhosis. Patients require more intensive monitoring and management as they are less likely to recover and readily progress towards multi-organ failure [5]. The main differences between decompensated liver cirrhosis and ACLF are the potential to recover and the progression towards multi-organ failure. Increasingly, ACLF is being considered as a discrete disease entity within liver cirrhosis or CLD [6]. The progression of liver disease is illustrated in Fig. 1.

### Assessment of underlying liver disease

The severity of the liver disease is assessed by clinical assessment, non-invasive imaging and biochemical blood tests known as liver function tests (transaminase blood tests, bilirubin, albumin and international normalised ratio (INR). Blood test results and clinical features are used to calculate composite risk scores that can predict the survival of patients. The two commonly used scoring systems are model for end-stage liver disease (MELD) and Child-Pugh score (CPS).

The MELD score is an objective assessment of patients with liver cirrhosis and is calculated using a combination of blood tests: creatinine, serum bilirubin and INR. The MELD score correlates to the severity of underlying CLD and predicts transplant-free survival [7, 8]; it can thus be used to stratify a patient’s need for liver transplantation (LT) within the next 3 months [8, 9]. MELD score is calculated from using this formulation: 9.5 × In [Creatinine (9 mg/dL)] + 3.78 × In [Bilirubin (mg/dL)] + 11.2 × In (INR)+ 6.43 [9]. The score ranged from minimum of 6 (mild disease) to maximum of 40 (severe disease) [10]. Patients with a score of 40 have a 300-fold increased risk of mortality waiting for a transplant compared to patients with a score of less than 12 [8, 11].

CPS is calculated from both objective parameters (serum bilirubin, INR or prothrombin time, serum albumin) and subjective parameters (clinical assessment of ascites and hepatic encephalopathy) [12, 13]. The score varies between 7 and 15, and the mortality is increased with a higher CPS [14]. The parameters for scoring Child-Pugh are mentioned in Table 2.

**Table 2:** Child-Pugh scoring system

	Parameters				
	Bilirubin (umol/L)	Albumin (g/dL)	Prothrombin time (in seconds)	Ascites	Hepatic Encephalopathy
1 point	<34	>35	<4	None	None
2 points	34–50	28–35	4–6	Mild	Grades I–II (or suppressed with medication)
3 points	>50	<28	>6	Moderate to severe	Grades III–IV (or refractory to medication)

Child A, points (5–6), 1-year mortality 100 %; child B, points (7–9), 1-year mortality 81 %; child C, points (10–15), 1-year mortality 45 %

The standard of care for patients with CLD (including ACLF) revolves around the management of the aforementioned complications of decompensation. There are currently no disease modifying therapies to reverse or resolve cirrhosis, currently the only definitive treatment is LT, the provision of which is limited by shortage of donor organs, long waiting times, peri-operative complications, transplant-associated morbidities such as rejection and immunosuppression, and the allied financial costs [15, 16]. As a result, the evaluation of novel therapies to improve outcome in patients with CLD is needed. Emergent therapies include infusing patients with stem cells to promote liver regeneration and subsequently improving liver function and fibrosis.

### Information on the intervention

#### Stem cells

Stem cells are undifferentiated cells that are able to proliferate in an effectively unlimited fashion [17]. There are two broad types of stem cells with therapeutic potential: embryonic stem cells and adult stem cells [18]. Embryonic stem cells (ESCs) can differentiate into any type of stem cells, but adult stem cells are less versatile in their differentiation. Stem cells can be obtained from self (autologous) or from a donor (allogenic) which can then be used for therapy. For this review, the main focus will be on adult stem cells. Mobilisation of stem cells from the bone marrow to the peripheral circulation can be induced with injection of a glycoprotein known as granulocyte colony-stimulating factor (GCSF).

Research has focused on bone marrow-derived stem cells in recent years due to ease of harvesting. Harvested cells can be infused into the patient in a number of ways, and these include infusion via peripheral vein [19] or centrally to the liver via the hepatic artery [20] or portal vein [21].

Several clinical studies have examined the effects of stem cell therapies in patients with CLD/ACLF [19–22]. These range from early proof of concept studies [23–25] through to larger randomised controlled trials (RCTs) [26, 27]. The results of these studies suggest that cell therapy is safe with no significant adverse events and has beneficial effects on CLD.

#### HSC

Haematopoietic stem cells (HSCs) are the only cells within the haematopoietic system that possess the potential for both multi-potency (ability to differentiate into many cell lines) and self-renewal (the ability to give rise to identical daughter HSCs without differentiation) [28]. HSC are commonly isolated from the blood, bone marrow, umbilical cord blood or occasionally from peripheral blood on the basis of their expression of specific surface markers as in CD34+ and CD133+ [19, 29, 30]. Other approaches include the use of unsorted peripheral blood mononuclear cells which contain HSC amongst other cell populations [31].

#### MSC

Mesenchymal stem cell (MSC; also called mesenchymal stromal cells) is a subset of non-haematopoietic stem cells [32]. MSC can be obtained from the blood and bone marrow, but they are also abundant elsewhere such as the skin, umbilical cord blood, adipose tissue, gut, lung, placenta, amniotic fluid, tendon, synovial fluid, skeletal muscle, liver and heart [33, 34]. MSCs have the potential to differentiate into hepatocytes, reduce hepatocyte apoptosis, increase hepatocyte regeneration, and reduce liver fibrosis by suppressing inflammatory responses [35]. In this review, MSC obtained from any source that fits the inclusion criteria will be included.

#### Unsorted stem cells

Stem cells that are neither haematopoietic or mesenchymal in nature will be classified under unsorted stem cells. These cell types will include bone marrow mononuclear cells (BM-MNCs) or bone marrow stem cells (BMSCs).

#### Granulocyte colony stimulating factor (GCSF)

GCSF is a growth factor that stimulates bone marrow to produce a large amount of stem cells and release them into the peripheral blood [36]. GCSF therapy is not only commonly administered prior to HSC cell harvesting but also has been investigated as an independent treatment option on its own has been investigated as a treatment option for CLD [37]. The common side effects of GCSF include flu-like illness, bone pain, fluid retention and abdominal discomfort or pain due to enlargement of the spleen [36]. GCSF is used in mobilising HSC into the circulation from where they can be isolated for clinical use. GCSF is not used in the isolation of MSC.

## Rationale for this review

Scoping searches performed on Cochrane Library (Cochrane Database of Systematic Review, Database of Abstracts of Reviews of Effects, Health Technology Assessments) and PROSPERO (up to 30/05/2015) identified five published systematic reviews of cell therapy in liver disease [26, 27, 38–40] and no in-progress reviews. Across the five reviews, none were without limitations. Major and common issues include basic or unclear search strategies, analysis of different study designs together, analysis of MSC and HSC therapies together and lack of clarity on limitations of (or reason for not) conducting meta-analysis or subgroup analysis. It will be prudent to perform new review because there have been few new original studies which fit the inclusion criteria since the last review in 2015 [41] as well as the many limitations of the current published previous systematic reviews which mentioned above. Depending on the availability and nature of the existing evidence subgroup analyses based on type of stem cell, route of administration and patient’s underlying liver conditions will be undertaken.

## Methods/design

### Aims and objectives

The aim of this systematic review is to evaluate the clinical effectiveness of cell therapies in the treatment of patients with CLD or ACLF. As ACLF is considered a discrete disease entity within CLD, it is a pre-specified subgroup for this review. Furthermore, as far as possible, CLD without ACLF will also be a discrete subgroup.

The proposed systematic review will answer the following main question: when compared to standard therapy what is the clinical effectiveness of (a) HSC, (b) MSC, (c) unsorted stem cells and (d) GCSF therapy in the treatment of CLD or ACLF?

Standard systematic review methodology aimed at minimising bias will be employed. Where data allows, the intention is to consider, through subgroup analysis, the evidence of effect in different underlying disease populations (as in viral hepatitis- or alcohol-related liver diseases), the effect of each type of stem cells (HSC, MSC, unsorted stem cell or GCSF therapy alone), the source of the stem cells (autologous and allogeneic stem cells) and the route of administration of the cells such as peripheral or central route.

Determination of comparative effectiveness between cell types and routes of administration will be considered if there are direct comparisons in studies included in the reviews. In addition, the potential for indirect adjusted comparisons will be assessed [42].

### Type of studies

Controlled trials will be included with no restrictions on the type of design.

All observational evidence will be obtained, whether controlled or uncontrolled, in order to gain an overview of existing observational evidence. Uncontrolled observational studies will be used where primary outcomes are not reported in the controlled studies or where uncontrolled studies have longer follow-up for these outcomes.

Existing systematic reviews will be selected in order to identify any primary studies that were not identified by the searches.

### Types of participants

Inclusion criteria:

Adult patients (≥18 years old) with

1. CLD
2. ACLF

Exclusion criteria:

1. Patient with acute liver failure (no evidence of liver cirrhosis)
2. Patient with cancer (unable to ascertain the effect of stem cells on tumour pathogenesis)

Studies on mixed populations of those defined under inclusion and exclusion criteria will only be included where the data for CLD or ACLF is presented separately.

### Types of interventions

1. Treatment with HSC of any dose, duration and mode of delivery with standard medical therapy with or without GCSF therapy to mobilise stem cells for collection/harvesting
2. Treatment with MSC from any source, any dose, duration and mode of delivery with standard medical therapy
3. Treatment with unsorted stem cells (BMSC and/or BM-MNC) of any dose, duration and mode of delivery with standard medical therapy with or without GCSF
4. Treatment with GCSF therapy only (without stem cell infusion) of any dose and duration with standard medical therapy

### Comparator

For studies where a comparator arm is included, comparators may consist of placebo, standard medical therapy or another treatment intervention listed above under interventions.

### Types of outcome measures

There will be no restriction placed on the type of clinical outcomes or the duration of follow-up for study selection to capture the additional evidence of adverse events occurring close to the time of stem cell infusion or GCSF injection. To guide data extraction and analysis, primary and secondary outcomes will be:

Primary outcomes:

1. Overall patient survival
2. Liver transplant-free survival
3. MELD
4. Quality of life
5. Adverse events specific to the intervention

Secondary outcomes:

1. Liver function tests
2. CPS
3. Events of liver decompensation as defined and reported by the study authors

## Search strategy

Cell therapy in liver diseases was first investigated in clinical phase studies in early 2000s, and hence, the searches will be run from year 1990 onwards. The following databases will be searched to capture both published and unpublished studies.

1. Bibliographic databases—MEDLINE, MEDLINE in Process and EMBASE, Cochrane Library CENTRAL database for published studies and additionally for systematic reviews the Cochrane Library Database of Systematic Reviews, Health Technology Assessment database and the Database of Abstracts of Reviews of Effects
2. The International Standard Randomised Controlled Trial Number (ISRCTN) database, United Kingdom Clinical Research Network (UKCRN), WHO International Clinical Trials Registry Platform (WHO ICTRP) Portal and ClinicalTrials.gov for ongoing studies
3. Hand searching of conference reports from the following databases between January 2012 and December 2015: the European Association for the study of Liver Disease, American association for the study of liver disease, Asian-Pacific association for liver disease, British association for the study of liver disease and British society of gastroenterology
4. Screening of citation lists of included studies and relevant systematic reviews

The searches of bibliographic databases will employ a combination of text words and index terms relating to liver disease and cell therapy as appropriate. There will be no language restrictions applied to the searches. Study design filters will not be used. A sample strategy for MEDLINE is provided in Appendix.

Search results will be entered into electronic database (ENDNOTE version X7.0.2 Thomson Reuters) to facilitate record keeping, duplicate removal, study selection and document writing.

## Data collection and analysis

### Selection of studies

To remove irrelevant articles, one reviewer will screen all the titles and abstracts, and to ensure consistency, another reviewer will check a proportion (minimum 50 % of all articles) independently. This way of screening articles is a limitation of the study due to this project being unfunded.

Hard copies of relevant articles will be acquired and assessed independently against the inclusion criteria by two reviewers. Discrepancies between reviewers will be resolved by discussion and by referring to a third reviewer if required. Full-text selection will be performed by two reviewers independently. Where necessary, translation (full/part) of non-English language articles will be undertaken to facilitate this process and subsequent reviewing. Where translations are not possible and this limits selection and/or reviewing, this will be reported. The study selection process will be illustrated using a PRISMA flow diagram.

### Data extraction and management

Data extraction of the included studies will be performed using a standardised data extraction form by one reviewer and checked independently by a second reviewer for all the studies. Disagreements will be resolved through discussion or referral to a third reviewer. For each study, the data required on (but not limited to) the following will be sought:

1. *Study characteristics*: authors, geographical origin, year of publication, study design (to include bias/confounding minimisation), years and duration of recruitment, number of arms, sample size and duration of follow-up
2. *Participant characteristics*: enrolment criteria, age, sex, number of participants, diagnosis and disease manifestations
3. *Intervention and comparator details*: sample size for each treatment arm, dose and type of interventions/comparator (HSC, MSC, unsorted stem cell or GCSF therapy alone), type of treatment received before or during therapy and the duration of treatment
4. *Results*: outcomes measured, time points, method of assessment, completeness of follow-up, statistical methods employed, findings, effect sizes and associated uncertainty

There are likely to be a limited number of RCT on this topic, and therefore, as mentioned previously, all observational evidence will be obtained, whether controlled or uncontrolled, in order to gain an overview of existing observational evidence. However, the uncontrolled observational studies will only be analysed where primary outcomes are not reported in the controlled studies or where uncontrolled studies have longer follow-up for these outcomes. To facilitate this decision-making and to be efficient, data from controlled studies will be extracted first and data from uncontrolled studies will only be extracted initially to determine design, population, intervention, outcomes and duration of follow-up.

## Assessment of risk of bias of included studies

Data will be extracted to allow quality assessment of the included studies. Study quality will be assessed using tools specific to a given study design. The risk of bias tool from the *Cochrane Handbook* will be used for RCTs [43]. For non-RCT studies, the domains in the risk of bias tool for RCTs can be used as a minimum assessment (accepting that the studies are not randomised).

For controlled observational studies, the guidelines outlined in Chapter 13 of the *Cochrane Handbook* will be followed [43]. The most relevant criteria for assessment in this area are likely to relate to how the groups were selected, differences in patient characteristics, loss to follow-up and biases and confounding in outcome assessment. Quality assessment for uncontrolled studies will be based on the guidance in the Centre for Reviews and Dissemination Handbook [44]. Items for consideration will include selection of patients (criteria and whether a consecutive series), detail on those lost to follow-up, use of objective and/or blinded outcome assessment.

## Analysis

Initially, a narrative synthesis of evidence will be undertaken. This will structure each intervention comparison relevant to the aims of the review (HSC vs usual care; MSC vs usual care; unsorted stem cells vs usual care; GCSF vs usual care) and by outcome and by population (CLD/ACLF). There will also be stratification by each study design contributing evidence. Subgroup analysis will be considered to investigate data on each type of stem cells, the source of stem cells (allogeneic and autologous) and the route of administration (central or peripheral infusion),

Data are likely to be presented using different outcome statistics, for example, mean difference, relative risk, and hazard ratio. Time points of reporting outcomes are also likely to vary across studies. Time points of 3 months or longer will be preferentially analysed to reflect the requirement for data on longer term survival and liver function. However, shorter term data (<3 months) will not be ignored as it is likely to relate to underlying population risk and procedure-related events. The events will be analysed as per following time points: 0–3 months, 3–12 months and beyond 12 months. There will be no time limit for outcomes such as adverse events and mortality.

Analysis methods will be guided by the considerations outlined in the Cochrane Handbook [43]. Meta-analytic methods will be employed where appropriate, to combine data for each population, comparison, outcome combination across the same or very similar time points. Summary statistics will most likely be pooled relative risk for dichotomous outcomes, pooled mean difference for continuous outcomes or pooled hazard ratios for time to event data. This may involve conversion of different statistics into a single, consistent measure, where appropriate assumptions are met, for example, by using the method of Parmar to obtain hazard ratios from dichotomous data [45]. Standardised mean differences will be considered if the same outcome is measured using different assessment tools (e.g. quality of life).

Appropriateness of performing meta-analysis and whether a fixed or random effects model is the most suitable will be determined by assessment of clinical and methodological heterogeneity rather than tests of heterogeneity from a fixed effects model [46]. The percentage of the total variability in the data due to between-study heterogeneity (*I*^2^ statistic) will be reported. Evidence from differing study designs (e.g. RCTs and observational studies) will not be quantitatively combined, but presented separately. It is likely that the random effects model will be the most appropriate for all analyses due to the underlying heterogeneity. The likelihood of publication bias will be investigated through the construction of funnel plots and appropriate statistical tests for small-study effects for each analysis of primary outcomes where 10 or more studies contribute data [43, 47].

The potential for sensitivity analysis of meta-analysis conclusion will be considered, for example, where there is a clear difference in methodological quality between studies of a similar design contributing data to a specific analysis.

As several interventions are considered in this review, the potential for undertaking adjusted indirect comparisons/multiple treatment comparisons will be explored, for example, where there are RCTs on different types of stem cell interventions with a common comparator (for example, HSC vs usual care and MSC vs usual care). The ability to undertake such analyses will be dependent on a number of key assumptions (e.g. the homogeneity, similarity and consistency assumptions) [48–50].

The findings of each analysis (effect size and precision) will be considered in conjunction with the methodological quality of the contributing studies, the variation in effect between studies and the importance of the outcome measures. The generalisability of findings will be discussed.

## Reporting of data

The review and its findings will be reported in accordance with the Preferred Reporting Items for Systematic Reviews and Meta-Analysis guidelines [51].

## Discussion

Liver cirrhosis is a significant cause of mortality worldwide for which there is no effective therapy except OLT. However, due to shortage of donor organs, many patients die whilst waiting for a LT. Hence, cell therapies have been studied as an alternative treatment option although their clinical effectiveness is still unclear. The aim of this systematic review is to address whether cell therapies (or GCSF alone) are effective interventions for the treatment of CLD and/or ACLF and if the method of harvesting stem cells or the route of their administration are effect modifiers. The findings will be of great interest to clinicians, healthcare decision makers and patients, and given the emergent nature of the interventions, it will also inform future research.

## Abbreviations

ACLF, acute on chronic liver failure; BM-MNC, bone marrow mononuclear stem cell; BMSC, bone marrow stem cell; CLD, chronic liver disease; CPS, Child-Pugh score; GCSF, granulocyte colony-stimulating factor; HCC, hepatocellular carcinoma; HSC, haematopoietic stem cells; INR, international normalised ratio; LT, liver transplant/transplantation; MSC, mesenchymal stem cells; MELD, model for end-stage liver disease; RCT, randomised controlled trial

## Appendix

Database: Ovid MEDLINE(R) <1946 to December week 3 2015>

Search Strategy:

1. exp Acute-On-Chronic Liver Failure/or exp Liver Diseases/or exp Liver Regeneration/or exp End Stage Liver Disease/or exp Liver Cirrhosis/
2. liver disease$.ti,ab.
3. liver cirrhosis.ti,ab.
4. acute-on-chronic liver failure.ti,ab.
5. liver regenerat$.ti,ab.
6. exp Hematopoietic Stem Cells/or exp Stem Cells/or exp Stem Cell Transplantation/or exp Bone Marrow/
7. exp Mesenchymal Stromal Cells/
8. stem cell$.ti,ab.
9. exp Granulocyte Colony-Stimulating Factor/
10. granulocyte colony stimulating factor.ti,ab.
11. mesenchymal stromal cell.ti,ab.
12. 1 or 2 or 3 or 4 or 5
13. 6 or 7 or 8 or 9 or 10 or 11
14. 12 and 13
15. limit 14 to (humans and yr = "1990 -Current")

## Additional file

Additional file 1: PRISMA-P (Preferred Reporting Items for Systematic review and Meta-Analysis Protocols) 2015 checklist: recommended items to address in a systematic review protocol*. (DOC 82 kb)
